# Multi-Area, Multi-Service and Multi-Tier Edge-Cloud Continuum Planning

**DOI:** 10.3390/s25133949

**Published:** 2025-06-25

**Authors:** Anargyros J. Roumeliotis, Efstratios Myritzis, Evangelos Kosmatos, Konstantinos V. Katsaros, Angelos J. Amditis

**Affiliations:** Institute of Communication and Computer Systems (ICCS), National Technical University of Athens, GR-157 80 Athens, Greece; stratis.myritzis@iccs.gr (E.M.); vangelis.kosmatos@iccs.gr (E.K.); k.katsaros@iccs.gr (K.V.K.);

**Keywords:** multiple areas and services and tiers, edge computing, artificial intelligence, optimization, inference

## Abstract

This paper presents the optimal planning of multi-area, multi-service, and multi-tier edge–cloud environments. The goal is to evaluate the regional deployment of the compute continuum, i.e., the type and number of processing devices, their pairing with a specific tier and task among different areas subject to processing, rate, and latency requirements. Different offline compute continuum planning approaches are investigated and detailed analysis related to various design choices is depicted. We study one scheme using all tasks at once and two others using smaller task batches. The latter both iterative schemes finish once all task groups have been traversed. Group-based approaches are presented as dealing with potentially excessive execution times for real-world sized problems. Solutions are provided for continuum planning using both direct complex and simpler, faster methods. Results show that processing all tasks simultaneously yields better performance but requires longer execution, while medium-sized batches achieve good performance faster. Thus, the batch-oriented schemes are capable of handling larger problem sizes. Moreover, the task selection strategy in group-based schemes influences the performance. A more detailed analysis is performed in the latter case, and different clustering methods are also considered. Based on our simulations, random selection of tasks in group-based approaches achieves better performance in most cases.

## 1. Introduction

Cloud and edge computing form a computing continuum (CC), combining the cloud’s scalability with the edge’s low latency and energy efficiency, optimizing data management for a more responsive system. Recent research indicates a trend toward edge-based data processing, which is particularly beneficial for advancing the Internet of Things (IoT) [1], as it can lower communication and storage costs and reduce energy consumption under appropriate resource scheduling. The growth of edge nodes represents a shift to decentralized data storage and processing, positioning computing resources closer to users via devices like mobile, local, and in-vehicle systems. This approach reduces data transmission needs, driving increased global investment in edge computing, reaching EUR 190 billion in 2023, a 13.1% rise from 2022, with forecasts to hit EUR 289 billion by 2026, and edge computing is expected to complement cloud computing across most enterprises by 2025 [2].

The expanding presence of the IoT and 5G have fueled a growing need for efficient data communication and processing. Edge computing has emerged as a solution by bringing services and functionalities traditionally hosted in the cloud closer to users. However, the effective utilization of the edge–cloud system remains a complex challenge due to the dynamic and resource-limited nature of it, with the resource allocation influenced by factors such as changing radio channel conditions, available computational resources, and device location. Optimally leveraging the edge–cloud system based on specific needs, similar to current work, requires collaborative scheduling [3,4,5,6], constituting a key area of focus in the literature.

In a multi-service and multi-tier CC architecture, tasks originating from various end devices (EDs) are processed through a hierarchy of processing layers (PLs) and computing devices (CDs). This processing spans from the ‘extreme edge’—comprising low-power platforms like unmanned aerial vehicles (UAVs)—to the centralized and high-performance cloud infrastructure, which is typically located away from the point of service demand. Between these endpoints, intermediate layers such as the ‘far edge’ (e.g., 5G base stations) and the ‘near edge’ (e.g., local aggregation hubs) dynamically manage task execution based on real-time resource availability and latency constraints. In our modeling, the multi-area dimension is added to the CC concept where the processing layers can be distributed across multiple areas including diverse users and services. Each area has its own extreme and far edges, which are aggregated into a shared near edge that supports all areas before finally connecting to the cloud for broader, centralized processing.

This work examines a multi-area, multi-service, and multi-tier CC system for optimizing regional infrastructure planning, including geographically distributed areas, with users having different processing needs. We assume that the regional network connection already exists. Our mechanism can be used by an infrastructure’s service provider (SP), such as the region’s (including multiple areas) local authority, to optimally design the CC system. Thus, following notation in [7], in the current work, an offline approach is provided to plan the CC system based on estimates of end devices and services within geographically distributed areas.

This approach uses tasks’ estimates, processing them at once for pre-deployment analysis, allowing allocation of resources to meet regional anticipated needs. The proposed schemes can be periodically executed at regular intervals, based on the specific needs of the devices in the region. Furthermore, we have to note that ‘services’ and ‘tasks’ refer to the same concept—computational functions or applications that are processed across edge and cloud resources within the continuum. Finally, in our paper, task refers to units of computation allocated in a one-shot, offline manner. There is no consideration of job sequencing or execution order. All tasks are known in advance and assigned simultaneously across edge–cloud resources. Hence, the focus lies entirely on optimal resource allocation, making sequencing analysis outside the scope of this study.

The edge–cloud system planning is based on the appropriate execution of tasks in specific processing devices in different tiers and areas; thus, the corresponding modeling includes the use of binary variables and the proposed mechanism utilizes binary nonlinear programming (BINLP) optimization to define the optimal configuration of computing devices within the CC. This involves determining the type, number, and location of devices across edge–cloud layers to execute tasks efficiently while minimizing infrastructure costs (other factors, like power usage, could also be examined without changing the problem’s structure), considering constraints on resources, rate, and tasks’ latency.

The proposed BINLP framework tackles a pressing real-world challenge: efficient resource allocation in complex, distributed digital ecosystems. From smart cities to remote healthcare and autonomous vehicles, modern services rely on heterogeneous devices and multi-tier networks. For instance, smart cities must manage diverse workloads like traffic control and environmental monitoring using edge and cloud infrastructure, while telemedicine systems require timely processing of patient data in rural regions. Misallocation of resources in such settings can cause service delays or even critical failures. Our model captures similar constraints realistically, assigning tasks across computing layers without oversimplifying the problem. Unlike generic approaches, it accounts for infrastructure cost, capacity limits, and service heterogeneity. By integrating spatial, architectural, and application-level considerations, our optimization method supports scalable and adaptive resource management. This makes it a practical solution for improving performance and reliability in real-world edge–cloud environments, where intelligent allocation is vital for operational success and sustainability.

The main contributions of our work are presented below:

- Planning of a regional edge–cloud system, where multiple services from various end devices across multiple areas, multiple types of processing nodes, and multiple CC tiers coexist in the same concept. The type of computing nodes, their number, and their allocation in the CC based on service’s requirements and network capacity is chosen. We model the CC system as a hierarchical tree-based system where services’ request flow is one-way, directed from end devices to the cloud.
- Two strategies are proposed to manage the computational complexity of the method that processes all tasks simultaneously (referred to as Full-Batch). A batch-based approach (assuming two different heuristics), configuring resources iteratively for smaller task groups, and a per-task allocation method that optimizes resources individually, enhances efficiency, and reduces time complexity in resource configuration. For the batch-based approaches two different concepts are provided: (a) the Large-Batch framework that has ‘memory’ of already used processing devices from previously allocated tasks and (b) the memoryless Small-Batch framework adding new processing devices to execute the tasks of the current batch.
- Unlike the Full-Batch scheme, which considers all tasks simultaneously, the other methods depend on the order of the tasks. The importance of task selection in each group is shown through the comparison between different ordering approaches, some of which are K-means and Agglomerative clustering methods. Based on the simulations, random task ordering provides better results in most cases. This could be explained by the fact that random selection of tasks better reflects the overall task mixture compared to fixed selection rules, leading to improved performance in batch-based approaches.
- Finally, service providers responsible for designing the system can use the proposed schemes to plan the compute continuum offline. Our findings help guide key design choices—such as the type of optimization strategy, the number of tasks processed at once, and their order. Specifically, our strategies highlight the trade-off between solution quality and computational efficiency. The Full-Batch approach, which processes all tasks together, offers the best performance but requires significant resources. In contrast, group-based schemes are more time-efficient and still effective—especially with medium-sized groups. Additionally, random task ordering often leads to better results, likely because each batch maintains a distribution similar to the original task set. These insights suggest promising directions for addressing the complex offline task planning in the edge–cloud environments, including the selection of batch’s size and tasks’ ordering.

The remainder of this work is organized as follows: in Section 2 the description of the related literature is presented, while in Section 3 the system’s scenario and the considered problem formulation and heuristic approaches are described in detail. In Section 4, the corresponding results are provided, while in Section 5, the current work is concluded.

## 2. Related Work

The importance of CC planning makes it an active studied area with much scientific interest [8,9,10,11,12,13,14,15,16]. CC planning is mathematically formulated as a mixed-integer non-linear programming (MINLP) problem, where tasks must be efficiently allocated to the edge–cloud layers while considering various constraints such as latency and computational capacity and minimizing a specific objective. However, for large scale planning, solving MINLP directly can be unachievable within a reasonable time frame, becoming computationally intractable. To address this, approximation methods, including machine learning (ML) or other heuristic approaches, are explored to achieve near-optimal solutions more efficiently. When it comes to ML approaches, not only is there no guarantee that the ‘learned’ solutions satisfies the MINLP constraints, but these methods also require substantial computational resources for model training and hyperparameter tuning. Additionally, when the input parameters or problem characteristics change, the machine learning model would need to be retrained from scratch. This occurs because there is no guarantee that previously learned solutions will work for the new problem instance. In contrast, the current work focuses on scalable solution strategies that directly address the MINLP as its size increases.

An indicative list of the above mentioned schemes is provided below. Conceptually, our hierarchical architecture draws inspiration from [11], where the authors examine the benefits of this hierarchical architecture compared to the conventional flat edge–cloud continuum for managing peak demand, and also propose appropriate algorithms for workload placement. In [12], the authors investigate how tasks can be distributed during UAV-assisted aerial inspections of power transmission infrastructure, leveraging a layered setup that includes an edge device, an intermediary hub (such as a smartphone or laptop), and a cloud server. They formulate the problem as a binary integer linear program aimed at optimizing either latency or energy usage, subject to the processing limitations of each device. The proposed approach is validated through deployment in a real-world application. Additionally, in [13] a hierarchical edge–cloud framework aimed at enhancing the deployment efficiency of cloud-native applications by decomposing them into micro-services is explored. To address this, the authors develop a mixed-integer linear programming (MILP) model focused on reducing both operational cost and service latency. Complementary to the MILP solution, they introduce a greedy heuristic and a reinforcement learning method to balance optimization quality with computational efficiency.

Authors in [14,15] assume that services are related to artificial intelligence (AI)-tasks, similar to our case where we validate the process in this type of tasks in Section 4. In [14] they investigate task allocation in edge–cloud infrastructure for augmented reality (AR) applications to minimize both user latency and mobile device energy costs and employ a heuristic solution based on the hill climbing algorithm to address an integer linear programming optimization problem. Authors in [15] study AI-related tasks with single-hop connections between mobile AR devices and edge or cloud servers, optimizing latency and accuracy by adjusting device–server assignments and frame resolutions. A block coordinate descent algorithm is used, utilizing AI model functions to correlate image resolution with the necessary floating-point operations for video analysis.

Furthermore, in [16] a resource allocation problem for distributed machine learning (DML) algorithms and applications that process continuous data is addressed. This framework models different ML and algorithmic instances originated by devices producing data continuously, over an infinite time horizon, processed by an algorithm running in edge and/or cloud layers. For this two-tier scenario, authors propose an integer linear programming (ILP) algorithm, considering requirements such as resource needs, accuracy, and delay, and a dual-objective, including optimizing cost and computation accuracy. A greedy algorithm, and one based on simulated annealing algorithm, are used to solve the resource allocation.

In the case of the offline approach of CC planning, presented in current work, there are no stringent time constraints. Thus, there is no need for real time decisions in terms of resource allocation, making the optimization theory an ideal and easy-to-use approach for our context. However, AI has widely been proposed for resource allocation in the edge–cloud context, such as in [17,18,19], and is instrumental for online resource allocation scenarios where rapid responses are essential for a CC system that continuously changes, a concept that is out of scope in presented work. Specifically, in previously mentioned related to AI works, deep reinforcement learning (DRL) is applied as a powerful approach for online decision making, handling high-dimensional state information and adapting to complex, dynamic environments, outperforming traditional heuristic methods.

Generally, in the related literature, the type and number of processing devices in each CC layer are predetermined. This is part of the CC deployment’s solution in the current work, i.e., the type and number of CDs are included in our outcome. While numerous existing studies—such as [12,13,14,16]—rely on linear programming (LP) frameworks for optimization, the present work introduces a more intricate, non-linear formulation. This complexity arises from the necessity to determine the required number of processing devices capable of handling diverse tasks originating from multiple EDs across several geographic areas. As a result, the problem incorporates minimization expressions not only in the objective function but also within its constraints, thereby evolving into a significantly more demanding BINLP model. Additionally, our framework extends the CC concept by incorporating a multi-area dimension, providing a broader perspective through the modeling of a regional (multi-area) CC system.

The work most closely related to the present study is that of [20], where a multi-tier and multi-service CC framework is analyzed. In that approach, an optimal solution is compared against a basic heuristic aimed primarily at generating feasible solutions. The comparison of both schemes focuses on the cost of proposed infrastructure and execution time. In contrast, the current work addresses a significantly more complex problem incorporating multiple geographic areas. Additionally, we present a variety of heuristic strategies designed to manage the increased complexity as the optimization problem scales. This aspect is not addressed in [20]. Furthermore, an in-depth analysis of task ordering is provided, offering a comprehensive examination of the broader system design and behavior.

## 3. System Model

This section includes all the relevant details about the modeling and assumptions of the considered system and the detailed analysis of problem’s formulation. Moreover, the mathematical notations along with the corresponding definitions are summarized in Table 1 to enable the readers to easily understand our models’ structure. The term ‘Known’ indicates predetermined parameters, while ‘To be computed’ refers to values derived from the optimization problem’s decision variables.

### 3.1. Scenario Setup

The general structure includes *L* PLs of processing units. These PLs are the extreme, far, near, and cloud layers for L=4 and Loc=1 (Loc parameter defines the number of PLs, except for the extreme layer, in each area). Each *a*th area has its own extreme ($PL_{a,1}$) and far ($PL_{a,2}$) layers and all the areas are aggregated to the near ($PL_{3}$) layer and then to the cloud ($PL_{4}$) layer. Moreover, we assume that there is existent connectivity support with specific data rates in communication links from edge to cloud layers.

**Figure 1:**
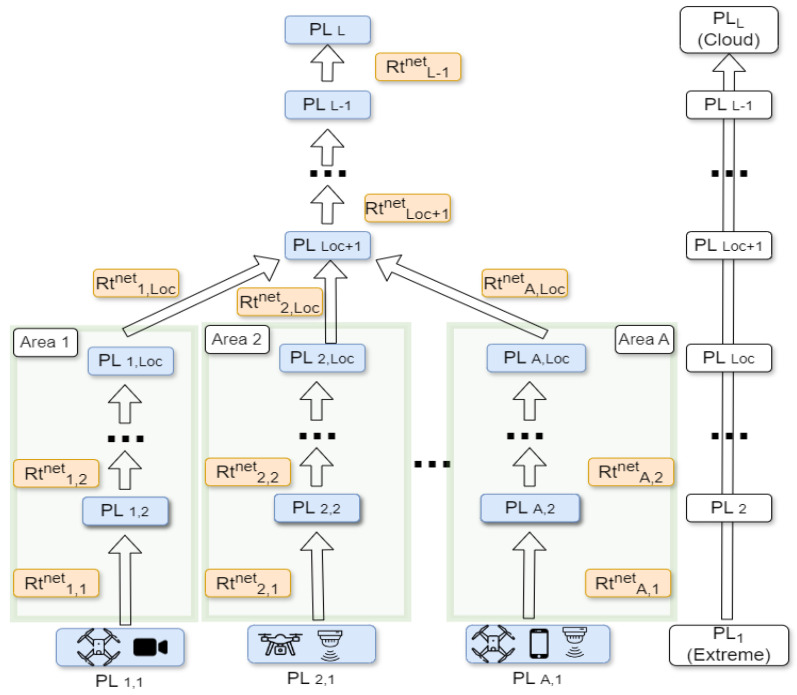
Multi-area, multi-service and multi-tier compute continuum configuration.

The whole configuration is presented in Figure 1 and we determine six different sets of parameters that characterize the end-to-end (E2E) architecture and are included in the optimization approach. Specifically, the sets of parameters are as follows: **(a)** Dev end devices (e.g., drones, cameras) which support **(b)** *S* different services related to specific tasks (e.g., object detection) and exist in **(c)** *A* areas (having data rate $Rt_{dsa}^{ED}$); **(d)** the links’ capacity $Rt_{z}^{net}$ and transmission latency ($l_{sza}^{net}$) of networks connecting the (z−1)th and *z*th processing layers. These parameters depend on the area and transferred task, i.e., more demanding tasks such as a 4K video upload could have a larger latency than a HD video upload; **(e)** *N* different types of computation devices for the processing of tasks (e.g., Raspberry Pi, NVIDIA, PowerEdge) and **(f)** L=4 processing layers (i.e., extreme, far, near, and cloud). Moreover, *j*th, with 1≤j≤N, processing device has a different processing (inference) latency ($l_{sj}^{comp}$), CPU ($R_{sj}^{CPU}$), and GPU ($R_{sj}^{GPU}$) resource consumption (%) among the services.

Finally, it is reasonable to assume that different types of computing devices are distributed across various processing layers within the edge–cloud continuum. For example, lightweight devices such as Raspberry Pi units are typically positioned closer to the end-user side—e.g., at the extreme or far edge—while high-performance systems like NVIDIA A100 GPUs or Dell PowerEdge servers are more suited to layers closer to the cloud, such as the near edge. Thus to model this logic we use the binary CL matrix of L×N dimensions where 1 indicates that the *j*th CD can be used in *z*th CC layer, explicitly modeling the fact that not all devices are suitable for all processing layers.

In the considered scenario, the *d*th, with 1≤d≤Dev, end device in *a*th area, with 1≤a≤A, can have one or more *s*th, with 1≤s≤S, services described by the binary DSA matrix of Dev×S×A dimensions with 1 meaning that *d*th ED in *a*th area requires the *s*th service. Also the network capacity of communication links ($Rt^{net}$) is predetermined and our problem includes the network part, assuming both the latency and data rate of communication hops between the CC levels.

In terms of latency, the *s*th service of *d*th ED in *a*th area has a maximum tolerable service latency $L_{dsa}^{req}$ and total latency $L_{dsa}^{tot}=L_{dsa}^{net}+L_{dsa}^{comp}$, including the network latency ($L_{dsa}^{net}$) and the processing latency ($L_{dsa}^{comp}$) at the CD similar to [15].

### 3.2. Problem Formulation

The general problem is shown below and the details about it follow after the mathematical structure:

(1) $$\begin{array}{c} \min_{f\in \{0,1\}}\sum_{j=1}^{N}\left[\sum_{a=1}^{A}\sum_{z=1}^{Loc}t_{zja}+\sum_{z=Loc+1}^{L}o_{zj}\right]\cos t_{j}\\ s.t.\sum_{w=1}^{A}\sum_{z=1}^{L}\sum_{r=1}^{S}\sum_{q=1}^{Dev}\sum_{j=1}^{N}f_{qrwdsazj}=DSA_{dsa}\left(C1\right)\\ f_{qrwdsazj}\leq CL_{zj}\left(C2\right)\\ \sum_{w=1}^{A}\sum_{j=1}^{N}\sum_{q=1}^{Dev}\sum_{r=1}^{S}\min(\sum_{s=1}^{S}f_{qrwdsa1j},1)\leq 1\left(C3\right)\\ \sum_{a=1}^{A}\sum_{d=1}^{Dev}\sum_{s=1}^{S}f_{qrwdsazj}R_{sj}^{CPU}\leq 100(C4)\\ \sum_{a=1}^{A}\sum_{d=1}^{Dev}\sum_{s=1}^{S}f_{qrwdsazj}R_{sj}^{GPU}\leq 100\left(C5\right)\\ L_{dsa}^{tot}\leq L_{dsa}^{req}\left(C6\right)\\ \sum_{d=1}^{Dev}\sum_{s=1}^{S}\left(1-\sum_{u=1}^{z}x_{dsau}\right)Rt_{dsa}^{ED}\leq Rt_{az}^{net}\left(C7\right)\\ \sum_{d=1}^{Dev}\sum_{s=1}^{S}\sum_{a=1}^{A}\left(1-\sum_{u=1}^{z}x_{dsau}\right)Rt_{dsa}^{ED}\leq Rt_{z}^{net}\left(C8\right) \end{array}$$

where the d,s,a,q,r,w,z,j indexes’ ranges for all the constraints are shown below:

(2) $$\begin{array}{c} (C1,C6):\forall 1\leq d\leq Dev,1\leq s\leq S,1\leq a\leq A,\\ (C2):\forall 1\leq q,d\leq Dev,1\leq r,s\leq S,1\leq a,w\leq A,\\ 1\leq z\leq L,1\leq j\leq N,\\ (C3):\forall 1\leq d\leq Dev,1\leq a\leq A,\\ (C4,C5):\forall 1\leq q\leq Dev,1\leq r\leq S,1\leq w\leq A,\\ 1\leq z\leq L,1\leq j\leq N,\\ (C7):\forall 1\leq a\leq A,1\leq z\leq Loc,\\ (C8):\forall Loc<z<L \end{array}$$

The information that is used in the considered problem can be described briefly with appropriate tuples as follows: **(a)** $\left\{L_{dsa}^{tot},DS_{dsa}\right\}$ for the relation among end devices and services of different areas, **(b)** $\left\{l_{sza}^{net},Rt^{net},Rt_{dsa}^{ED}\right\}$ for the system’s connectivity part, **(c)** $\left\{R_{sj}^{CPU},R_{sj}^{GPU},\cos t_{j},l_{sj}^{comp}\right\}$ for the relation among computing devices and services, and **(d)** $CL_{zj}$ for the allocation of processing devices in CC.

The aforementioned analysis results in optimization problem formulation (called as “Full-Batch” because all system’s tasks are considered simultaneously as one batch), which is summarized in (1) with constraints shown in (2). This problem belongs to integer optimization, widely known for the high solution’s time complexity [21].

**Variables of the problem**: $f_{qrwdsazj}$ (binary) means that the *s*th task of *d*th ED is served to *z*th level by (qrwj)th device if $f_{qrwdsazj}=1$. This problem’s design allows the fact that each end device’s *s*th task in *a*th area can be served, if this results in better performance, in a different processing device of the same *j*th type. Afterwards the maximum number of *j*th type of computing devices that are possible to be used are Dev×S×A, and in that hypothetical scenario, none of the rest of the devices will be allocated.

To conclude, we have added the q,r,w dimensions in *f* variable, replicating the *d*th device, *s*th service, and *a*th area respectively; to be possible, the same *j*th computing device is to be used at most Dev×S×A times. In that scenario each service’s task will be allocated to another device of *j*th type.

An illustrative example follows, inspired by Section 4.1, to better clarify the index functionality. Consider a region with two domains: the first is an agricultural zone (i.e., *a* = 1) where a drone (i.e., *d* = 1) performs precision farming through crop health monitoring using object detection (i.e., *s* = 1) services. The second domain represents a village environment (i.e., *a* = 2) with elderly residents who have household robots (i.e., *d* = 2) in their homes. These robots assist with daily activities through monitoring body posture by pose detection (i.e., *s* = 2), encouraging people to stand up after prolonged periods of inactivity. Finally, regarding processing devices, a Raspberry Pi (i.e., *j* = 1) and a GPU (i.e., *j* = 2) are considered. Based on this information, we determine the problem parameters as Dev = 2, *S* = 2, *A* = 2 and *N* = 2.

Upon solving the problem, the binary solution is stored in the *f* variable. We present a simplified solution example to demonstrate how results are interpreted. As output from the BINLP solver, suppose the drone’s object detection task is assigned to a GPU in the near processing layer, i.e., $f_{00011132}$ = 1, while the robot’s pose detection task is allocated to a Raspberry Pi in the far layer, i.e., $f_{00022221}$ = 1.

**Objective of the problem**: The objective function includes the total cost of used processing devices with $cost_{j}$ to be the *j*th device’s cost. Specifically, $t_{zja}$ (3) and $o_{zj}$ (4) express the number of *j*th type of processing devices, the first one for z≤Loc in each area and the latter for the z>Loc, where the areas are aggregated after the Loc layer.

The internal minimization functions in both (3) and (4) have been used to avoid adding the same processing device multiple times, considering the fact that one CD can serve various tasks originated by different end devices. Particularly, for the first branch in (3), we consider the case that in the extreme edge of each area one CD can be installed to each ED. In terms of the second branch, multiple services originated by different EDs in a specific area can be allocated to the CDs in upper layers of that area. For higher CC layers (z>Loc) in (4), the services from all areas are aggregated.

Hence, min formula for z = 1 includes only the summation across services because the analysis in the extreme layer is performed per ED. For z>Loc all services are considered and the d, s, a indexes are included in the min function, while in the intermediate case, the summation across areas is excluded from the min part, because this branch presents each area’s conditions.

(3) $$t_{zja}=\left\{\begin{array}{c} \sum_{w=1}^{A}\sum_{d=1}^{Dev}\sum_{r=1}^{S}\sum_{q=1}^{Dev}\min(\sum_{s=1}^{S}f_{qrwdsa1j},1),\;for\;z=1\\ \sum_{w=1}^{A}\sum_{r=1}^{S}\sum_{q=1}^{Dev}\min(\sum_{d=1}^{Dev}\sum_{s=1}^{S}f_{qrwdsazj},1),\;for\;1<z\leq Loc \end{array}\right.$$

(4) $$o_{zj}=\sum_{w=1}^{A}\sum_{r=1}^{S}\sum_{q=1}^{Dev}\min(\sum_{d=1}^{Dev}\sum_{s=1}^{S}\sum_{a=1}^{A}f_{qrwdsazj},1),\;for\;Loc<z$$

(5) $$L_{dsa}^{net}=\sum_{z=1}^{L-1}\left[x_{dsaz}\sum_{u=1}^{z}l_{sua}^{net}\right]$$

(6) $$L_{dsa}^{comp}=\sum_{w=1}^{A}\sum_{z=1}^{L}\sum_{j=1}^{N}\sum_{q=1}^{Dev}\sum_{r=1}^{S}\left[f_{qrwdsazj}l_{sj}^{comp}\right]$$

**Processing constraints (C1–C5)**: For the ED that has a specific service, this has to be served at a specific CC level and by one processing device (C1). The processing device has to be appropriate for usage in the selected processing layer (C2) and the allocated tasks cannot overpass the resources consumption of that device (C4), (C5). The resources are provided in %, hence the maximum available resource is 100%, denoted on right side of (C4), (C5).

Additionally, more or different resources can be used, e.g., storage, related to the specific scenario, without changing the problem’s formulation, just the number of constraints. Considering that the EDs are of small size and processing power, we allow at most one processing device to be paired with them at z=1 layer (C3). This constraint can be easily reformulated to allow each end device to be matched with multiple processing devices by substituting 1 with the desired number of assignments.

**Latency constraint (C6)**: The left part includes total latency of each service that consists the network and computation latencies provided in (5) and (6), respectively. Particularly, our analysis is based on the hierarchical tree based system [11]. Thus, the total latency is the summation of communication hops’ latencies plus the computation latency of CD where the task is executed.

The communication latency in (5) includes the $l_{sza}^{net}$ latency summarized from the 1st to *z*th hop (through the *u* index) in which the d,s,a task is finally executed. The substitution described in (7) and used in (5) has been made to present clearly (5). Particularly, $x_{dsaz}$ expresses the CC layer that each service’s task of an ED in a specific area will be executed.

(7) $$x_{dsaz}=\sum_{w=1}^{A}\sum_{r=1}^{S}\sum_{q=1}^{Dev}\sum_{j=1}^{N}f_{qrwdsazj}$$

**Communication links’ rate constraints (C7, C8)**: Each link (orange boxes in Figure 1) has a specific predetermined rate threshold and the total rate of not executed transferred tasks, across this link, cannot overpass this value. To appropriately capture the tasks that have not yet been executed, we use the formula $\left(1-\sum_{u=1}^{z}x_{dsau}\right)$ which is 1 if the (*d*, *s*, *a*) task has not been executed till *z*th layer. The *x* parameter is described in (7).

Furthermore, the rates are modeled as $Rt_{az}^{net}$ in (C7) for the links, until the Loc level, that are different for each area. Finally, for the higher layers, where the areas’ rates are aggregated, the $Rt_{z}^{net}$ in (C8) is used.

### 3.3. Heuristic Approaches

In the worst case, all areas’ EDs have all services and the executed tasks are T=Dev×S×A. The total possible CDs for tasks’ allocation are Y=T×L×N, considering that *j*th CD can be allocated to any CC layer, and each task can be assigned to a distinct CD of *j*th type. A brute force allocation examines all the $Y^{T}$ possibilities, filtering the valid options, subject to constraints, to find the best objective’s solution. In this example the high time complexity of the studied integer problem and its dependence on Dev, *S*, and *A* parameters are, intuitively, highlighted.

Specifically, similar to [16], our problem can be viewed as a more complex version of the bin packing problem. In the bin packing problem, there are as many bins (here, computing devices) with a common capacity (here, CPU and GPU capacities) as necessary. The goal is to find the fewest (here, minimizing the total cost of CC planning) that will hold all the items (here, tasks). Since the bin packing problem is NP-complete and serves as a simplified version of our problem, it follows that our problem is also NP-complete.

Assuming the computational time of our problem, two heuristic approaches are introduced, reducing *Dev*, *S*, and *A* parameters’ size by breaking the Full-Batch problem into smaller, sequential optimization problems. The (*d*, *s*, *a*) tasks are divided into batches and the “Large/Small-” Batch optimization methods are applied to each batch. Consequently we iteratively solve smaller problems to simplify the overall process until all the system’s tasks are considered.

These heuristic solutions are based on appropriate matrix adaptations, ensuring an objective mathematical structure free from subjective elements. Unlike other approximation schemes for combinatorial optimization problems, our method offers significant advantages. Approaches such as simulated annealing or machine learning require extensive parameter tuning, with effectiveness varying widely based on parameter selection. In contrast, our method remains stable, consistent, and adaptable across different scenarios.

Since no universal parameter selection exists, subjective choices can lead to drastically different results. In contrast, our structured methodologies provide a stable and adaptable framework that can be seamlessly integrated (in a ‘plug and play’ way) into open-source or commercial solvers, such as the Gurobi solver, ensuring robust and reliable optimization across a wide range of applications.

(8) $$\begin{array}{c} \min_{f\in \{0,1\}}\sum_{q=1}^{Dev}\sum_{r=1}^{S}\sum_{w=1}^{A}\sum_{j=1}^{N}\left[\sum_{z=1}^{Loc}\left[{\mathrm{cost}}_{qrwzj}\sum_{a=1}^{A_{S}}t_{qrwzja}\right]+\sum_{z=Loc+1}^{L}o_{qrwzj}{\mathrm{cost}}_{qrwzj}\right]\\ s.t.\sum_{w=1}^{A}\sum_{z=1}^{L}\sum_{r=1}^{S}\sum_{q=1}^{Dev}\sum_{j=1}^{N}f_{qrwdsazj}=DSA_{dsa}\;\left(C1\right)\\ f_{qrwdsazj}\leq CL_{qrwdazj}\;\left(C2\right)\\ \sum_{w=1}^{A}\sum_{j=1}^{N}\sum_{q=1}^{Dev}\sum_{r=1}^{S}\min(\sum_{s=1}^{S_{S}}f_{qrwdsa1j},1)\leq 1\;\left(C3\right)\\ \sum_{a=1}^{A_{S}}\sum_{d=1}^{D_{S}}\sum_{s=1}^{S_{S}}f_{qrwdsazj}R_{sj}^{CPU}\leq R_{qrwzj}^{CPU}\;\left(C4\right)\\ \sum_{a=1}^{A_{S}}\sum_{d=1}^{D_{S}}\sum_{s=1}^{S_{S}}f_{qrwdsazj}R_{sj}^{GPU}\leq R_{qrwzj}^{GPU}\;\left(C5\right) \end{array}$$

**Small-Batch:** For each batch the problem in (1) is solved considering only the end devices, services, and areas included in the batch. Thus the reduced sets $D_{S}$ (in *d*, *q* dimensions), $S_{S}$ (in *s*, *r* dimensions), and $A_{S}$ (in *a*, *w* dimensions) are assumed instead of the initial *Dev*, *S*, and *A* sets. The compute continuum’s processing devices and layers are kept the same, hence the *N* and *L* sets in *j* and *z* dimensions, respectively, remain constant. Additionally, the available link rates are reduced based on the previously allocated tasks.

Moreover, to guarantee the feasibility of the solution of the initial problem, an extra constraint is assumed. Particularly, in the *i*th batch the (C3) constraint (allowing at most one computing device to each end device in the extreme layer of current batch) already exists. However, a CD can be allocated to an ED, while this ED already has another CD (serving one or more of its tasks in previous batches). This can result in more CDs allocated to one ED.

Thus the extra constraint $\sum_{w=1}^{A_{S}}\sum_{r=1}^{S_{S}}\sum_{q=1}^{D_{S}}\sum_{j=1}^{N}f_{qrwdsa1j}\leq P_{da}$ guarantees that no extra processing device can be allocated to an end device that has already been served in extreme layer in a previous batch. Especially, we initially assume the *P* matrix of $D_{S}\times A_{S}$ dimensions with all ones and for the *d*th ED in *a*th area with an allocated CD we set $P_{da}=0$. In that way at most one CD can be allocated to one ED in an area. Hence the solution of initial problem remains feasible after each batch’s execution. Generally, the solution for *i*th batch adds appropriate computing devices in the edge–cloud continuum to cover the tasks of that batch.

**Large-Batch:** This approach solves, in each batch, the problem in (1), knowing in parallel the processing devices that have already been allocated in previous batches, resulting in the problem described in (8). To incorporate this ‘memory’ of previously allocated computing devices into the optimization problem, the decision variable f has *q*, *r*, and *w* dimensions with the initial sizes, i.e., Dev, *S*, and *A* respectively. In this way, after each iteration, the information of already used CDs from the processing pool is collected. Additionally, the dimensions *d*, *s*, and *a* correspond to the $D_{S}$, $S_{S}$, and $A_{S}$ sets, respectively, similar to the Small-Batch scheme. The current mechanism is termed as ‘Large-Batch’ because it employs larger decision variable matrices compared to the Small-Batch. However, the matrix dimensions *z* and *j* have lengths of *N* and *L*, respectively, as in the Small-Batch scheme, because the types of computing devices and the number of processing layers remain unchanged.

Thus, in the *i*th batch, we know the CDs’ remaining resources ($R^{CPU}$, $R^{GPU}$) in (C4), (C5), starting from 100% initial resources. In each batch, the resources of the paired computing devices are reduced based on the tasks assigned to them. After solving the problem in (8), the updated resource states are carried forward and used in the formulation of the (i+1)th batch.

In terms of the CL matrix used in constraint (C2), the same logic as the one in the Full-Batch mechanism is followed. It models the valid allocation of CDs to the continuum layers, explicitly accounting for the fact that not all devices are compatible with all processing layers. To ensure that the overall solution remains feasible across multiple batches, we introduce an additional requirement. When a CD is allocated to the *d*th edge device in the *a*th area in the extreme layer, we block any further allocations of different CDs to the same (d,a) pair in subsequent batches. This is achieved by setting the corresponding positions in the CL matrix to zero, effectively forbidding those allocations.

This mechanism prevents the issue observed in the Small-Batch approach, where multiple CDs could be incorrectly assigned to the same ED in the extreme layer due to batch-wise optimization. In that way we ensure that the feasibility of the initial problem is preserved throughout the entire batch sequence.

Finally, (C6), (C7), (C8) constraints of (1) are the same in (8) assuming that *d*, *s*, *a* belong to $D_{S}$, $S_{S}$, $A_{S}$ reduced sets and the available link rates are reduced based on the previously allocated tasks. All constraints’ ranges are similar to (2), but with upper limits of *d*, *s*, *a*, *q*, *r*, *w*, *z*, *j* as described above.

In the objective of (8), the parameters t,o have a similar mathematical structure as in (3) and (4), respectively. Particularly, the parameter *t* is applied by retaining only the summations over the *d* and *s* dimensions compared to (3). Similarly, the parameter *o* is applied by keeping only the summations over the *d*, *s*, and *a* dimensions, in contrast to (4). In the current approach, the additional dimensions *q*, *r*, *w* are introduced without associated summations (unlike in (3), (4)) to explicitly model the ’memory’ aspect of the system. These dimensions allow the optimization model to retain information about the computing devices that have been selected in previous batches, along with their remaining resources. This enables our method to make informed allocation decisions based on the cumulative state of the system in each new batch.

In that way the Large-Batch can result in the usage of less CDs (hence less cost of CC planning) compared to the Small-Batch, because the latter continuously adds CDs to satisfy the tasks of the new batch.

## 4. Simulation Results and Discussion

The approaches presented in Section 3 are versatile and can be applied to various types of tasks. Without loss of generality we evaluate them by considering several AI-related tasks. The rapid advancement of AI and edge computing devices has given rise to the Edge AI [22,23] which involves performing AI computations near the network edge, a concept included in simulations below. Moreover, there is extensive benchmarking of AI model performance across different services and processing devices, as demonstrated in [24,25].

Therefore, optimally designing an edge–cloud computing architecture that supports various AI tasks [26] becomes crucial for meeting both user and network demands in the AI era. By leveraging benchmarked AI service information, such architectures can effectively balance computational requirements with resource constraints. This directly aligns with the simulation objectives of our work presented in the following sections.

### 4.1. Simulated Scenarios

To evaluate our schemes, a set of processing and network data generated from realistic scenarios in the EU Horizon XGain project (https://xgain-project.eu/) were used. These are described in XGain Deliverable D3.4: “System integration and dry-run testing feedback report (2nd version)” which will be made publicly available upon approval by EU reviewers. Specifically, in terms of processing the CPU and GPU consumption and inference latency of a Raspberry Pi 4 (RPI4), a Nvidia Jetson Xavier (Jet_Xavier) and a RTX system (including a personal computer (PC) with an Intel(R) Xeon(R) Gold 5218 CPU and a NVIDIA RTX 3090) under object detection (OD), pose detection (PD), and speech-to-text (S2T) tasks have been investigated. Especially inference latency ($l_{sj}^{comp}$ in (6)), CPU ($R_{sj}^{CPU}$ in (1)), and GPU ($R_{sj}^{GPU}$ in (1)) consumption exploit these values. To extend the processing pool in current simulated scenarios, we have also added A100, L4, and H100 NVIDIA GPUs and Jetson AGX Orin, resulting in the list of N=7 CDs.

Considering the trend that leverages progressively more powerful processing devices from the extreme edge to cloud, balancing lower inference times with higher costs and power consumption, we constructed the binary CL matrix of (1) and (8) as {RPI4, Jet_Orin, Jet_Xavier} in extreme and far and {RTX, A100, L4, H100} in near and cloud layers. To collect values for the extra CDs, except for the ‘already measured’ RPI4, Jet_Xavier, and RTX, we followed the simplified logical assumption that inference latency, CPU, and GPU consumption are related with the RTX (most powerful among the rest ‘known’ devices) through the factor of $TOPS_{RTX}/TOPS_{Extra}$ (Tera Operations per Second) with Extra = {Jet_Orin, A100, L4, H100} and TOPS collected for Jet_Orin and GPUs from the links in the ‘Source’ column listed in Table 2.

Table 2 summarizes the key information from this analysis, including the processing devices used, their allocation across the edge–cloud continuum layers, whether metrics are based on measured values or logical assumptions (related to the aforementioned TOPS analysis), and relevant source links for clarity. Additionally cost values based on Amazon and eBay were collected. It should be noted that the simulated scenarios demonstrate how the proposed schemes operate rather than focusing on precise real-world values for all devices. Our methods are dataset-independent and can accommodate different input values, as these serve merely as parameters to the algorithms. Users can substitute appropriate values for their specific use cases without modifying the universal model framework.

**Figure 2:**
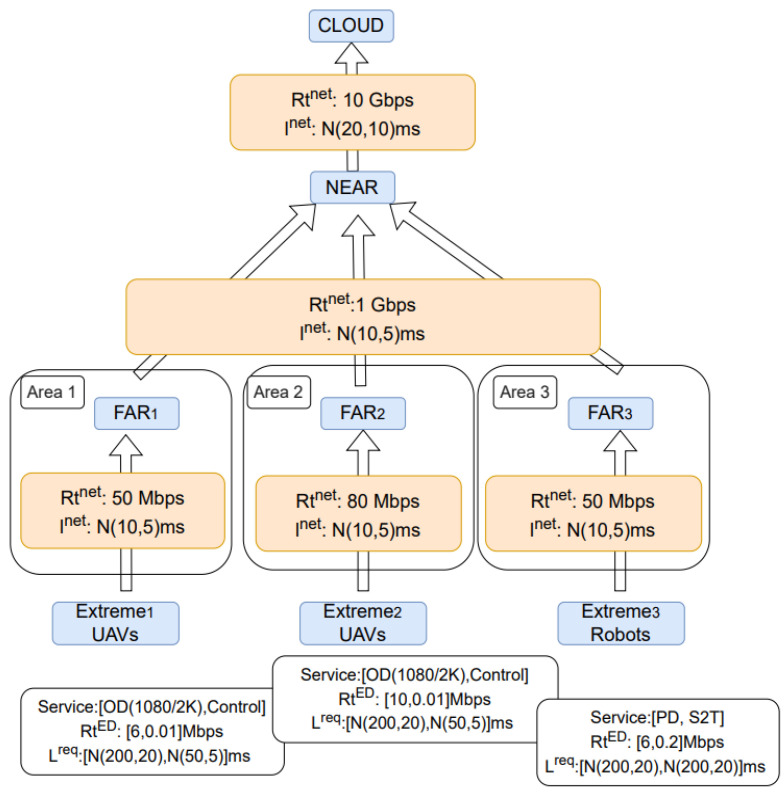
Scenario’s parameters including networks’ capacity and latency, EDs’ types per area, their services, rates, and tolerable latencies.

In terms of network data, in simulated scenarios, we use representative values of communication latency (modeled as Normal distribution) between the hops (Figure 2). Generally, this data is originated by values measured and provided in XGain D3.4, followed by the logical assumption for larger latency in te last hop towards cloud. Moreover, typical 5G uplink (UL) rates are assumed in the 1st hop, followed by 1 Gbps (fiber link) in the 2nd hop and 10 Gbps (Gigabyte Passive Optical Network (GPON)) for the 3rd hop.

Furthermore, an indicative set of XGain’s scenarios are assumed, including the UAVs’ object detection tasks and the household robots operating by vocal commands and monitoring the persons’ activities such as their pose detection. Particularly, in our simulated scenarios, 1st and 2nd areas could be agricultural areas where UAVs are used in precision farming to monitor crop health by capturing high-resolution images. These are translated to ‘1080p/2K’ OD and ‘control’ service types with UL rates and latencies matching each service and taking values as defined in [27] (pp. 21 and 51).

The 3rd area could be a village where household robots exist and their functionality is translated to S2T and PD services. The information of EDs and their services in each area is presented at the bottom of Figure 2, e.g., in Area 1 there are UAVs having OD service of 6 Mbps and N(200, 20) ms latency, and Control service of 10 Kbps and N(50, 5) ms latency. Finally, without loss of generality, we assume Dev = 8 EDs per area, resulting in scenarios with 24 EDs having 48 executed tasks.

Additionally, 15 different simulated scenarios, whose structure is shown in Figure 2, have been executed based on different samples of normal distributions and the average cost and time execution are found. Analysis focuses on trends rather than exact performance values, and proposed schemes can be applied directly to real measurements if available. Finally, optimization problems are solved in Python (version 3.10) by the Gurobi solver [28] and executions are performed in a PC including 11th Gen Intel(R) Core(TM) i7-11700 @ 2.50 GHz and 16 GB RAM.

### 4.2. Performance Analysis of the Proposed Schemes

**Figure 3:**
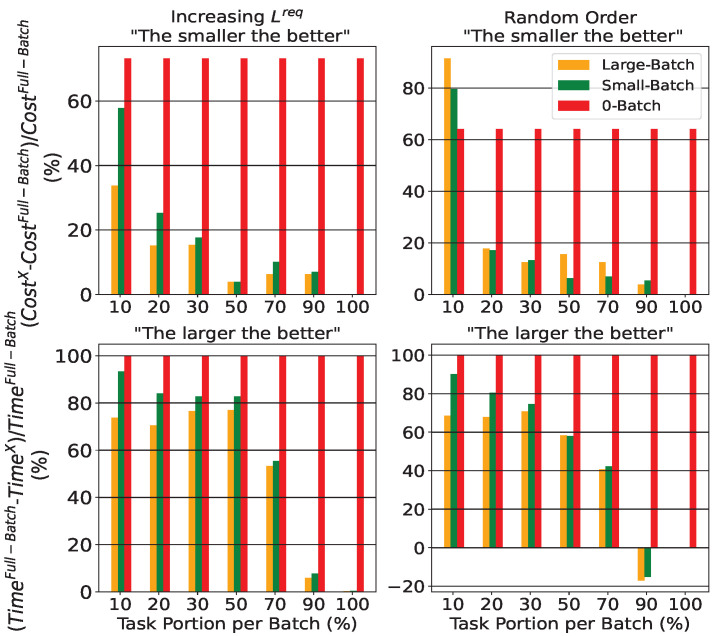
Percentage difference of system’s cost and time execution of Heuristic approaches from Full-Batch process.

Figure 3 presents the deviation in cost (top) and time execution (bottom) of Heuristic approaches (called *X* in *y*-axis) from the Full-Batch for two different task orderings ($L^{req}$ in left side and random in right side) and different task portion per batch (in *x*-axis of subfigures). Except for the described heuristic schemes in Section 3, the 0-Batch mechanism, where zero means the per-task process, i.e., there is no batch usage, is also examined. In this scheme each individual task is assigned to a CD in a CC layer to minimize processing costs and meet rate and latency needs, subject to the remaining CDs’ resources from the already paired tasks.

It is noteworthy that since execution time is hardware-dependent, we deliberately present time percentages rather than absolute values in terms of time execution of proposed schemes in Figure 3. This approach provides hardware-independent insights that focus on the relative performance of our mechanisms, offering valuable comparisons between the described approaches.

**Figure 4:**
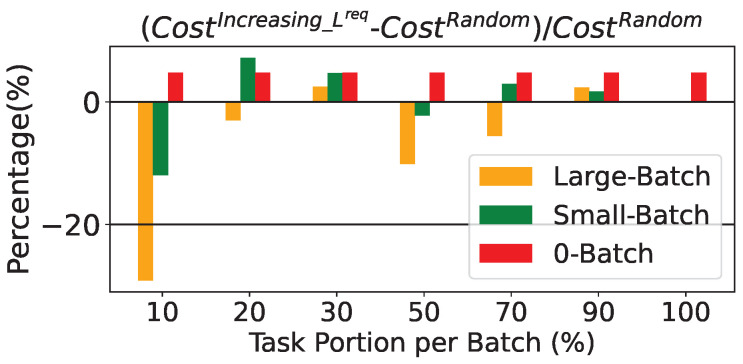
Percentage difference of Heuristic approaches’ system cost between the different tasks’ ordering.

The considered approaches iterate through batches made by tasks ordered by increasing $L^{req}$ or randomly ordered and the task selection influences the result of Large/Small/0-Batch schemes (described further in Figure 4). It is obvious that 0-Batch is the most time efficient, having nearly 100% time difference compared to the Full-Batch, but has the worst cost in general. Additionally the batch size influences the outcome of Large-Batch and Small-Batch mechanisms, while the remaining schemes are batch independent.

As the scenarios’ number of tasks grow, leading to large-scale problems, the design approach involves including fewer tasks in each batch. This reduces the computation time required for optimization schemes because less (*d*, *s*, *a*) pairs are active. However, smaller groups, provided to the group-based optimization schemes, may capture less information about the overall characteristics of the initial task pool. In contrast, the Full-Batch mechanism processes all tasks together as a single input, preserving their complete information, but this approach can be time-consuming and computationally expensive for large-scale scenarios.

In our simulations, the best performance is achieved for 30% and 50% tasks per batch (first row subfigures). Compared to the Full-Batch, there is a cost increase of at most about 20% (first row subfigures) with a time reduction of at least about 60% (second row subfigures). These observations are drawn from jointly analyzing the results of the Large/Small-Batch schemes (i.e., yellow and green columns, respectively) under both task orderings in the respective portions. As the batch size increases, each batch includes a larger portion of the total tasks. When the batch size reaches its maximum of 100%, all the tasks are considered simultaneously. Additionally, when all tasks are processed in a single batch (100% task portion), both Large-Batch and Small-Batch mechanisms achieve identical performance to Full-Batch, as indicated by the zero-height bars across all subfigures. This proves that both mechanisms have been correctly formulated.

Furthermore, in most cases, the Large-Batch scheme results in better performance compared with Small-Batch mechanism, but with higher time execution. This shows that the characteristic of ’memory’ in Large-Batch can help the planning, but results in larger execution time because of the larger matrices’ size compared to the ’memoryless’ Small-Batch approach. Moreover, Large/Small-Batch may lead to longer execution times than Full-Batch as batch size nears total tasks’ number (subfigures in second row). This is logical considering the batch iteration and the larger matrix sizes in each optimization iteration.

Finally the influence of task ordering is shown in Figure 4. We consider seven task portion groups, and for each group, we evaluate three different schemes: Large-Batch, Small-Batch, and 0-Batch, resulting in a total of 21 evaluation cases (i.e., 21 columns shown in Figure 4). For each of these three schemes, we run experiments using both increasing $L^{req}$ and random task orderings. This allows us to assess the impact of task selection strategy across all three schemes and seven batch sizes. As illustrated in Figure 4, in 15 out of the 21 cases (i.e., 15 columns with non-negative height), the random ordering outperforms (achieving a lower total cost) or has at least equal performance with the increasing $L^{req}$ approach. Furthermore, both ordering mechanisms have equal cost for (Large and Small)-Batch schemes in 100% task portion that is ordering independent. To conclude, the Full-Batch approach is independent of tasks’ ordering and does not appear in the figure.

The observed superiority of random task selection in each batch can be explained as follows: the best performance is achieved when all tasks are processed together (in Full-Batch scheme). To approximate this in batch-based approaches, it is advantageous to maintain a task distribution within each batch that mirrors the full set. Random selection supports this by naturally preserving task diversity and avoiding biases introduced by fixed or structured grouping methods, as the increasing $L^{req}$ approach. This approach follows the same underlying logic as data shuffling in machine learning, where data points are randomly reordered before being grouped into batches for training. This standard practice effectively reduces sampling bias while enhancing both model performance and training stability throughout machine learning processes.

### 4.3. Further Analysis of Task Ordering Schemes

To examine how task selection schemes affect compute continuum planning performance, we extend our analysis by comparing three approaches: random ordering (which outperformed increasing $L_{req}$ ordering in previous results), agglomerative clustering, and k-means clustering methods. The latter two methods have been chosen due to their popularity and significant difference in their underlying structure. The agglomerative clustering is a hierarchical, bottom-up method that iteratively merges data points or clusters based on similarity, forming a dendrogram to represent the hierarchical structure [29]. Additionally, the k-means clustering is a popular partitioning algorithm that divides data into clusters by iteratively assigning points to the nearest centroid and updating centroids based on the cluster mean until convergence [30].

Specifically, the goal is to group the initial set of tasks into meaningful clusters based on their computational and communicational similarities. Considering the analysis in Section 3, each (*d*, *s*, *a*) task is described by the tuple ($Rt^{ED}$, $L^{req}$, $R^{CPU}$, $R^{GPU}$, $l^{comp}$, $Rt^{net}$, $l^{net}$), where $Rt^{ED}$ and $L^{req}$ are the rate and latency requirements of the task; $R^{CPU}$, $R^{GPU}$ and $l^{comp}$ are the CPU, GPU, and inference time of the task averaged in all considered CDs, because these values only depend on the type of tasks; and $Rt^{net}$ includes the 1st and 2nd hops’ rate of *a*th area where the task belongs and $l^{net}$ includes the 1st and 2nd hops’ communication latency of *a*th area where the task belongs. These tuples are the inputs to clustering algorithms.

In the current subsection we investigate whether grouping tasks based on their similarity can enhance the effectiveness of CC planning. Once the clusters are formed, we employ a heuristic method to reorder the tasks in order to be grouped for executing the optimization schemes. We begin with the cluster having the smallest average Euclidean distance from its centroid, then proceed by selecting the next cluster whose centroid is closest to the previously considered cluster. This process continues until all clusters have been traversed. Through this approach, the tasks are reordered and combined into a single batch, which is then separated in groups based on tasks’ portion for the execution of the proposed schemes.

In Section 4.1, we analyzed scenarios with 48 tasks. Now, we examine scenarios with larger sets of tasks, specifically 70, 80, 90, and 100 tasks, across different simulation configurations. Particularly, 15 different simulated concepts are investigated for each of the four aforementioned sets of tasks and the average cost of edge–cloud continuum has been collected. Furthermore, a task portion of 30% per batch is considered as a representative case to evaluate medium-sized batches, which demonstrated good performance in Figure 3.

As the number of tasks increases, resulting in a larger number of active (*d*, *s*, *a*) pairs, the complexity of the optimization problems described in Section 3 also grows. Therefore, we focus our evaluation on the performance of the Small-Batch scheme, which is more computationally manageable in such scenarios. This is the most time efficient approach for large-scale problems compared to the rest optimization mechanisms. Specifically, we examine different task ordering methods—random, agglomerative, and k-means—within this scheme. Finally, the scikit-learn framework [31] has been applied for the clustering algorithms.

**Figure 5:**
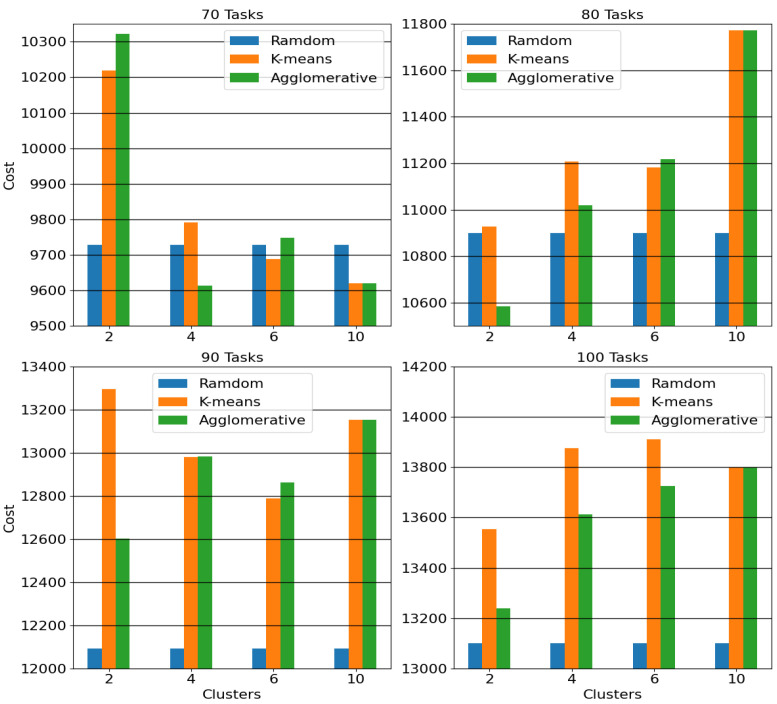
Small-Batch mechanism’s cost applying in different tasks’ ordering schemes under various number of clusters.

Figure 5 presents the cost of the Small-Batch scheme under the three aforementioned task ordering methods and varying numbers of clusters, i.e., 2, 4, 6, and 10 clusters. The performance of random ordering remains constant across the different number of clusters, because this method does not depend on tasks’ clustering. In most cases random task selection outperforms the other approaches (for 90 and 100 tasks, the random ordering is better for all the clusters’ groups), resulting in the lowest cost.

Consequently, based on these simulations, we conclude that grouping tasks based on their similarity does not significantly improve performance. Instead, random ordering, which better preserves the characteristics of the initial task pool in each group, leads to superior results. Finally, observing the performance among the k-means and agglomerative ordering, as the tasks increase, the agglomerative mechanism achieves better or at least similar results with the k-means, e.g., for 100 tasks in all clusters’ groups while in 90 tasks in three out of four groups.

To conclude our findings, the Full-Batch optimization method achieves the best performance but incurs a significantly higher execution time, while the 0-Batch approach is the most time efficient, but with the worst cost of CC planning. Moreover, the group-based approaches like Large-Batch and Small-Batch provide a more efficient trade-off between performance and computational cost. Large-Batch, which retains resource memory, generally outperforms Small-Batch but requires a longer execution time. For large-scale problems, Small-Batch remains the most time-efficient option. Additionally, task selection strategy is crucial, with random ordering consistently delivering superior results, especially in scenarios with a higher number of tasks.

## 5. Conclusions

This work introduces an optimal design for a multi-area, multi-service, and multi-tier compute continuum and proposes task group-based heuristics to address scenarios where the size of the initial problem leads to excessive execution times. Our heuristic solutions use matrix adaptations, ensuring an objective, consistent mathematical structure. Unlike other optimization methods that require extensive parameter tuning and scenario-specific adjustments, our approach integrates directly into commercial or open-source solvers. We provide a complete mathematical formulation that can be, without modification, implemented in these solvers to obtain optimal solutions. This seamless integration delivers robust and reliable optimization performance across diverse problem instances and scenarios. In particular, this study emphasizes scalable solution methods designed to effectively tackle the binary optimization problem as its size grows.

While multi-tier CC frameworks have been explored in the literature, our paper is novel in its comprehensive integration of multiple areas while simultaneously addressing the three key pillars (i.e., multiple services, tiers, and areas) of CC planning. The proposed schemes are well-suited for offline CC planning, where real-time decisions are not required. In contrast, for scenarios demanding rapid online decision-making, AI models can offer greater efficiency; however, substantial effort is required to train these models and ensure that the solutions they produce both satisfy the problem’s constraints and maintain quality across varying problem sizes.

Results show that the Full-Batch optimization problem, which considers all tasks as input, yields superior performance but at the cost of significantly higher execution time compared to the Large-Batch and Small-Batch group-based mechanisms. These group-based approaches achieve acceptable performance with reduced execution time (compared to the Full-Batch scheme) when applied to medium-sized task batches. Notably, the Large-Batch mechanism, which retains a “memory” for the remaining resources in CC, outperforms in most cases the “memoryless” Small-Batch scheme, albeit with increased execution time due to the inclusion of larger matrices in the computations. Hence, for very large scale problems, the Small-Batch approach can be the most time efficient to be executed. Lastly, task selection strategy in group-based schemes significantly impacts the results. To thoroughly assess its influence, we compare random task selection with clustering methods (i.e., k-means and agglomerative approaches) and non-clustering strategies (i.e., ordering by increasing $L^{req}$). In most cases, random tasks’ choice exhibits superior performance. This is obvious in scenarios with higher number of tasks.

From the perspective of the service provider, who has to conduct the offline CC planning, our proposed strategies can be adopted depending on the specific use case. Among the investigated models, the Full-Batch scheme is the most effective when there are no strict time or computational constraints. However, group-based schemes, while inferior in performance, offer greater time efficiency. If a group-based model is chosen, the size of each batch (i.e., the number of tasks per group) becomes a critical factor. Our findings suggest that medium-sized groups yield performance closer to the optimal Full-Batch scheme. Lastly, the task’s selection strategy is another key consideration. According to our results, a random selection of tasks can achieve better performance.

In future work, we plan to investigate advanced AI models capable of learning the characteristics of our proposed optimization schemes, with a strong emphasis on ensuring constraint satisfaction and robustness across varying problem sizes. This approach aims to facilitate fast, reliable decision-making for online resource allocation within the computing continuum. Additionally, we will conduct further research on task selection schemes to enhance the effectiveness of these approaches. 

## Figures and Tables

**Table 1:** Mathematical notation and definitions.

Symbol	Definition
**System Parameters (Known)**	
*L*	Number of processing layers in the cloud continuum
$PL_{a,z}$	Processing layer *z* in area *a*
Loc	Number of processing layers (except extreme layer) in each area
Dev	Number of end devices
*S*	Number of different services
*A*	Number of areas
*N*	Number of different types of computing devices
**Indices**	
*d*	Index for end devices, 1≤d≤Dev
*s*	Index for services, 1≤s≤S
*a*	Index for areas, 1≤a≤A
*j*	Index for computing device types, 1≤j≤N
*z*	Index for processing layers, 1≤z≤L
q,r,w	Replication indices for devices, services, and areas, respectively
**Decision Variables (To be computed)**	
$f_{qrwdsazj}$	Binary variable: 1 if *s* task of *d* ED is served at *z* level by (qrwj) device
$x_{dsaz}$	Binary indicator (based on *f*): 1 if task (d,s,a) is executed at layer *z*
**System Matrices (Known)**	
DSA	Binary matrix of dimensions Dev×S×A indicating service allocation
CL	Binary matrix of dimensions L×N for processing device-layer allocation
**Network Parameters**	
$Rt_{dsa}^{ED}$	Data rate from end device *d* for service *s* in area *a* (Known)
$Rt_{z}^{net}$	Network capacity of links connecting layers (z−1) and *z* (for z>Loc) (Known)
$Rt_{az}^{net}$	Network capacity for area *a* of links connecting layers (z−1) and *z* (for z≤Loc) (Known)
$l_{sza}^{net}$	Network transmission latency for service *s* connecting layers (z−1) and *z* in area *a* (Known)
$L_{dsa}^{net}$	Total network latency for task (d,s,a) (To be computed)
**Computing Resources and Latency**	
$l_{sj}^{comp}$	Processing (inference) latency for service *s* on device type *j* (Known)
$L_{dsa}^{comp}$	Total computation latency for task (d,s,a) (To be computed)
$R_{sj}^{CPU}$	CPU resource consumption (%) for service *s* on device type *j* (Known)
$R_{sj}^{GPU}$	GPU resource consumption (%) for service *s* on device type *j* (Known)
**Latency Requirements and Constraints**	
$L_{dsa}^{req}$	Maximum tolerable service latency for task (d,s,a) (Known)
$L_{dsa}^{tot}$	Total end-to-end latency (based on the solution *f*) for task (d,s,a) (To be computed)
**Objective Function**	
$cost_{j}$	Cost of computing device type *j* (Known)
$t_{zja}$	Number of type *j* devices used at layer *z* in area *a* (for z≤Loc) (To be computed)
$o_{zj}$	Number of type *j* devices used at layer *z* (for z>Loc) (To be computed)

**Table 2:** Computing devices information for simulated scenarios.

Devices	CC Layer	Metrics (Inference, CPU, GPU)	Source
Raspberry Pi 4	Extreme, Far	Measured	https://xgain-project.eu/
Nvidia Jetson Xavier	Extreme, Far	Measured	https://xgain-project.eu/
Nvidia Jetson AGX Orin	Extreme, Far	Related to $TOPS_{RTX}/TOPS_{Orin}$	https://developer.nvidia.com/embedded/downloads (accessed on 23 June 2025)
RTX System (PC + RTX 3090)	Near, Cloud	Measured	https://xgain-project.eu/
NVIDIA A100 GPU	Near, Cloud	Related to $TOPS_{RTX}/TOPS_{A100}$	https://www.nvidia.com/en-eu/data-center/a100/ (accessed on 23 June 2025)
NVIDIA L4 GPU	Near, Cloud	Related to $TOPS_{RTX}/TOPS_{L4}$	https://resources.nvidia.com/l/en-us-gpu (accessed on 23 June 2025)
NVIDIA H100 GPU	Near, Cloud	Related to $TOPS_{RTX}/TOPS_{H100}$	https://resources.nvidia.com/l/en-us-gpu (accessed on 23 June 2025)

## Data Availability

The original contributions presented in this study are included in the article. Further inquiries can be directed to the corresponding author.

## Abbreviations

The following abbreviations are used in this manuscript, presented in alphabetical order:

AI	Artificial intelligence
AR	Augmented reality
BINLP	Binary non-linear programming
CC	Compute continuum
CD	Compute device
DML	Distributed machine learning
DRL	Deep reinforcement learning
E2E	End-to-end
ED	End device
GPON	Gigabyte passive optical network
ILP	Integer linear programming
IoT	Internet of Things
ML	Machine learning
OD	Object detection
PC	Personal computer
PD	Pose detection
PL	Processing layer
S2T	Speech-to-text
SP	Service provider
TOPS	Tera operations per second
UAV	Unmanned aerial vehicle
UL	Uplink
